# The “Green Gold” May Have a Chance Towards Sustainability: *Persea americana* In Vitro Callus Cultures

**DOI:** 10.3390/biotech15030047

**Published:** 2026-06-26

**Authors:** Vanessa Dalla Costa, Raffaella Filippini

**Affiliations:** Department of Pharmaceutical and Pharmacological Sciences, University of Padova, Via Marzolo, 5, 35131 Padua, Italy; raffaella.filippini@unipd.it

**Keywords:** avocado, phenolics, antioxidant, secondary metabolites, nutritional analysis

## Abstract

Superfoods have gained increasing attention for their nutritional and functional properties, with avocado (*Persea americana* Mill.) among the most prominent examples owing to its health-promoting compounds. However, avocado cultivation is associated with several challenges, including high water demand, environmental impact, seasonal variability, and post-harvest losses. To address these limitations, in vitro plant cell cultures represent a sustainable and controlled alternative for producing avocado-derived material. In this study, avocado var. Hass callus cultures were established and evaluated as a potential source of functional metabolites. Colourimetric assays performed at different growth stages identified 14-day-old callus as the most enriched in phenolic compounds and antioxidant activity; this material was therefore selected for further analyses. LC–ESI–QTOF–MS/MS profiling revealed a phenolic-rich composition, including flavonoids, proanthocyanidins, galloyl derivatives and phenylpropanoid-related compounds, consistent with vegetative plant tissues. Nutritional analysis showed high moisture content and low lipid levels, differing in composition from the avocado pulp, along with a high content of attention-grabbing nutrients, such as protein and fibre. Overall, although further studies are required to confirm compound identity and assess safety for future applications, avocado calli represent a promising sustainable platform for the production of value-added bioactive compounds.

## 1. Introduction

In response to the increasing diet-related diseases, growing attention has been directed toward healthier dietary patterns. This shift has contributed to a surge in demand for functional foods, including so-called “superfoods”, which are valued not only for their nutritional composition but also for their perceived natural origin and associated health benefits [1]. Within this context, avocado (*Persea americana* Mill.), which has been appreciated for its health-promoting properties for centuries [2], is now widely recognised as a superfood with significant functional and therapeutic potential.

From a compositional and phytochemical perspective, avocado fruit is characterised by a complex and highly valuable biochemical profile that underpins its recognised health-promoting properties. The pulp is particularly rich in monounsaturated fatty acids (mainly oleic acid), along with polyunsaturated and saturated fatty acids, together with other bioactive compounds such as carotenoids, tocopherols, phytosterols (such as β-sitosterol), phenolic acids, flavonoids, and condensed tannins, which collectively contribute to its antioxidant and anti-inflammatory activities, including cardiovascular and metabolic diseases [2,3,4,5].

Along with the edible pulp, increasing attention has been directed toward the phytochemical composition of non-edible parts of the plant, such as seeds, peels, and leaves, which are often discarded despite their high biological value. Avocado by-products may account for up to 25–30% of the total fruit weight, representing a significant yet underutilised source of bioactive compounds [6]. Several studies have demonstrated that these tissues contain even higher concentrations of phenolic compounds than the pulp, highlighting their potential as alternative sources of functional ingredients. In particular, avocado seeds and peels are rich in phenolics, flavonoids, proanthocyanidins, and other bioactive compounds associated with antioxidant and antimicrobial properties [7,8,9,10]. Advanced analytical studies have further identified metabolites such as chlorogenic acid derivatives, catechins, quercetin glycosides, and procyanidins, supporting the functional value of these by-products [9,11,12]. Moreover, avocado leaves recently emerged as a promising source of phytochemicals such as phenolic compounds and flavonoids, with demonstrated antioxidant, anti-inflammatory, and potential anti-diabetic activities [13,14].

The increasing recognition of these nutritional and functional properties has been accompanied by a significant rise in avocado consumption worldwide. However, the growing demand for avocados and similar health-oriented foods has also led to a significant expansion in production, raising concerns about environmental and socio-economic impacts, particularly regarding resource use and sustainability. The expansion of avocado cultivation has led to significant deforestation and loss of ecosystem services, particularly in major producing regions such as Mexico. Additionally, its high water demand and intensive agricultural practices are increasing pressure on natural resources and contributing to environmental degradation [1,15,16,17].

From a reproductive standpoint, the species presents a protogynous dichogamous flowering system, which promotes cross-pollination but at the same time reduces pollination efficiency under suboptimal environmental conditions [18,19]. As a result, only a very small proportion of flowers develop into mature fruits, with extensive flower and fruitlet drop commonly observed. Moreover, the use of seed propagation leads to high genetic variability due to spontaneous hybridisation, making it difficult to maintain desirable traits and resulting in non-uniform orchards [19,20,21].

These limitations have driven increasing interest in alternative propagation strategies. In this regard, in vitro culture techniques offer promising opportunities, particularly for the development of controlled regeneration systems [22,23,24,25]. Among these, callus cultures have attracted attention mainly because they serve as a transitional stage toward somatic embryogenesis [23,24], which is one of the most effective approaches for clonal propagation.

Greater success has been achieved with immature zygotic embryos, which are more responsive and capable of producing embryogenic callus when cultured in the presence of strong auxins such as picloram or 2,4-D [23]. This type of callus is particularly important because it can give rise to somatic embryos, providing a basis for the regeneration of genetically uniform plants. This approach has been extensively investigated in avocado not only for propagation purposes but also as a platform for genetic improvement and transformation studies, despite the still limited efficiency and reproducibility of the protocols [20,22].

While in vitro culture techniques in avocado have been predominantly investigated as tools for plant regeneration and somatic embryogenesis, their potential extends beyond propagation, offering opportunities for the controlled production of plant biomass independent of environmental constraints. The possibility of using in vitro-derived material as an alternative food source [23,24,25] becomes particularly relevant in light of the increasing pressure on global food systems.

The combined effects of climate change, land degradation, water scarcity, and population growth are placing unprecedented pressure on conventional agricultural systems, limiting their capacity to meet the rising food demand [26,27].

In this context, the present study aims to investigate the potential of in vitro callus cultures of *Persea americana* var. Hass as a novel food source, focusing on the production of undifferentiated biomass for alimentary purposes. At the same time, increasing attention has been directed toward foods that provide not only nutritional value but also health-promoting effects, particularly those rich in bioactive compounds associated with antioxidant and protective activities [28,29,30]. For this reason, the obtained biomass was further characterised by analysing its phytochemical profile, including total phenolic and proanthocyanidin contents, and antioxidant activity.

## 2. Materials and Methods

### 2.1. Calli Obtainment

To highlight the best explant source for callus induction, preliminary experiments were done on different *Persea americana* materials obtained by Hass-variety fruits purchased on the market, and leaves from the Botanical Garden of Padova (Italy). The material—fruit peel, endosperm, and leaf—was cut into pieces and treated with different sterilisation times (Table 1).

After the sterilisation, the explants were placed on agarised media in dishes, and the materials’ responsiveness to the sterilisation process was followed. Table 2 shows the basal media and the plant growth regulator used in the preliminary experiments. All culture media were supplemented with 2,4-dichlorophenoxiacetic acid (2,4-D), naphtalenacetic acid (NAA), and kinetin (K), used either alone or in combination. Basal medium components, hormones, and agar were purchased from Duchefa (Micropoli, Milan, Italy).

Based on the responsiveness of the different explant types and their callogenesis induction efficiency, assessed as the proportion of explants forming callus, leaves were selected as the most suitable source material. To obtain a reliable supply of leaf explants, ripe avocado seeds were cleaned, suspended with toothpicks, and germinated hydroponically in tap water. The resulting plantlets provided the leaf material used for the subsequent callus induction trials. Once the plantlets reached the appropriate growth stage, the leaves were harvested and washed thoroughly with tap water containing detergent. The sterilisation process has been carried out by dipping the plant material in 80% ethanol (1 min), followed by 15% sodium hypochlorite with a wetting agent (7 min). The leaves were rinsed three times in sterile distilled water under a laminar flow cabinet. Leaf blades were cut into an area of almost 1 cm^2^ and placed half on MS and half on WPM media; the two media resulted in the best choice for leaf explant callogenesis induction. In the second induction experiment, leaf explants, prepared in the same way as above, were still placed on MS and WPM media along with MSA and B5A media, showing the plant growth regulator balance as reported in Table 3.

The explants, as well as the obtained biomass, were cultivated in a growth chamber at 25 ± 1 °C in a 16/8 h photoperiod, and subcultured every 6 weeks.

### 2.2. Chemical Analysis

All the chemical investigations were done on juices extracted by pressing the defrost-harvested calli with the aid of quartz powder and a pestle. Once a homogeneous consistency was achieved, samples were subjected to ultrasonic treatment for 40 min and subsequently centrifuged at 13,200 rpm. The resulting supernatants, juices, were collected for the analyses.

#### 2.2.1. Coulorimetric Analyses

Colourimetric analyses were performed on Hass juices extracted from calli harvested on the 14th (T14), 28th (T28), and 42nd (T42) days of growth. Total phenolic content (TPC) was evaluated using the Folin–Ciocalteu assay following the method described by Li et al. [35]. Gallic acid (Fluka, Buchs, Switzerland) served as the reference standard for calibration. Briefly, 200 µL of suitably diluted sample or standard solution was mixed with 1 mL of Folin–Ciocalteu reagent (Fluka, Buchs, Switzerland) diluted 1:10. After 4 min, 800 µL of saturated sodium carbonate solution (75 g/L) was added. The reaction mixture was incubated at room temperature for 30 min, then absorbance was measured at 765 nm using a HeλIOS spectrophotometer (Thermo Electron Corporation, Waltham, MA, USA). Calibration curves: y = 0.0435x + 0.0177 R^2^ = 0.9995 (gallic acid 1–25 µg/mL). Samples were analysed in duplicate; the results are reported as means ± standard deviation (SD).

Total proanthocyanidin content (TPcC) was quantified through the vanillin assay, adapted from the procedure reported by Truzzi et al. [36]. Catechin (Merck, Milan, Italy) was used as the standard compound for calibration. For the assay, 25 µL of appropriately prepared sample or standard solution was combined with 1 mL of 1% vanillin solution prepared in 70% H_2_SO_4_. After incubation at room temperature for 15 min, absorbance was recorded at 500 nm. Calibration curve: y = 0.1093x − 0.0008; R^2^ = 0.9997 (0.6–5 μg/mL). Samples were analysed in duplicate; the results are reported as means ± SD.

Antioxidant capacity was assessed using the DPPH radical scavenging assay according to Suriyaprom et al. [37]. Ascorbic acid (Sigma-Aldrich, Milan, Italy) was used as the positive control for calibration. In brief, 100 µL of appropriately diluted sample or positive control was added to 400 µL of DPPH solution (0.1 mM; Sigma-Aldrich) freshly prepared. Following a 30 min incubation period, absorbance was measured at 517 nm. Antioxidant activity was calculated as the percentage inhibition of DPPH discolouration caused by the presence of antioxidant compounds. Calibration curve: y = 11.768x − 0.9896; R^2^ = 0.9994 (0.8–6 μg/mL). The results, means ± SD of samples analysed in duplicate, are expressed as the percentage of inhibition of 6 μL of juices.

#### 2.2.2. LC-MS/MS Analysis

LC-MS/MS analysis was carried out on Hass juices extracted from calli harvested on the 14th day of the growth cycle. The analysis was carried out using an Agilent 1290 Infinity II liquid chromatography system coupled to an Agilent 6550 mass spectrometer (Agilent Technologies Inc., Santa Clara, CA, USA). Chromatographic separation was achieved on a Gemini C6-Phenyl column (5 μm, 250 × 4.6 mm; Phenomenex, Bologna, Italy) equipped with a matching guard column and maintained at 40 °C throughout the analysis. The mobile phase consisted of water containing 0.1% (*v*/*v*) formic acid (eluent A) and acetonitrile (eluent B). The optimised gradient elution program was set as follows: 0–8 min, 97% A; 8–26.5 min, 75% A; 26.5–40 min, 20% A; and 40–42 min, return to 97% A. The flow rate was maintained at 0.75 mL/min, while the injection volume was 5 μL. Chromatographic data were monitored at 265 and 325 nm, and UV–Vis spectra were collected within the 190–700 nm wavelength range. Mass spectrometric analyses were performed using a Dual AJS ESI ion source operating in negative ionisation mode. Source parameters were set as follows: drying gas temperature 300 °C with a flow rate of 5 L/min, sheath gas temperature 250 °C with a flow of 11 L/min, and nebuliser pressure of 35 psi. Capillary and fragmentor voltages were adjusted to 3500 V and 260 V, respectively. For MS/MS experiments, fragmentation patterns were acquired at collision energies of 0, 10, and 20 eV using an isolation window of 4 *m*/*z*. Instrument control and data acquisition were managed using MassHunter Workstation Data Acquisition 10.0 software (Agilent Technologies Inc., Santa Clara, CA, USA), whereas spectral processing and interpretation were performed with MassHunter Qualitative Analysis 10.0 (Agilent Technologies Inc., Santa Clara, CA, USA).

### 2.3. Nutritional Analysis

The nutritional composition was evaluated on fresh callus material by EPTANORD Food Analysis and Consulting (Conselve-Padova, Italy). Moisture, ash, and lipid contents were determined according to the ISTISAN 1996/34 protocol and expressed as g/100 g of fresh weight [38]. Protein concentration was assessed following the ISO 1871:2009 method and reported as g/100 g [39]. Dietary fibre content was quantified using the AOAC Official Method 985.29 (1986) and expressed as g/100 g [40]. Total carbohydrate content was calculated according to ISTISAN 1996/34 guidelines [38]. The energy value was estimated in accordance with Regulation (EU) No. 1169/2011 of 25 October 2011 and expressed as kcal/100 g. Lipid percentage was estimated by GC-FID according to MI 1634 rev 3 2023 [41].

### 2.4. Statistical Analysis

All results are presented as mean ± standard deviation (SD). Statistical analyses were carried out using GraphPad Prism software v 7.05 (GraphPad Software Inc., San Diego, CA, USA). Correlations between variables were analysed through Spearman correlation analysis and represented using scatter plots. Differences among the sampling times (T14, T28, and T42) were evaluated using the non-parametric Kruskal–Wallis test. Statistical significance was established at *p* < 0.05.

## 3. Results and Discussion

### 3.1. Callus Obtainment

As a preliminary step, different avocado explants were evaluated for their suitability for callus induction, including fruit peel, endosperm, and leaf tissues. Peel explants showed a very high contamination rate (90%). Moreover, the few non-contaminated explants rapidly developed browning symptoms and failed to produce callus, indicating that this tissue was not suitable for callogenesis under the tested conditions. Similarly, endosperm explants remained free from contamination but underwent rapid browning shortly after culture establishment and did not exhibit any callus formation. In contrast, leaf explants showed a markedly lower contamination rate (6%) and maintained their original green colouration throughout the initial culture period, without evident signs of tissue deterioration. Furthermore, leaf tissues displayed a greater capacity to remain viable under in vitro conditions, making them the most promising explant source among those tested. Based on these preliminary observations, leaves were selected as the starting material for the establishment of undifferentiated avocado cell cultures.

The callogenetic response of leaf explants cultured on eight different media is reported in Table 4.

Marked differences in callus induction efficiency were observed in the explants tested in the eight media. Among them, MS and WPM proved to be the most callogenic media, exhibiting the highest frequencies of callus induction and the best overall callus development. Consequently, these two media were selected for subsequent experiments using leaves from hydroponically grown plants as starting material.

Table 5 summarises leaf explant survival after sterilisation and the percentage of callogenesis success in the media tested in the two experiments performed. In this case, the sterilisation time was reduced from 10 to 7 min, as the leaves exhibited a less coriaceous texture than those obtained from mature trees growing in the Botanical Garden. This adjustment was made to minimise potential tissue damage caused by prolonged exposure to sterilising agents while maintaining adequate decontamination efficiency. The softer texture of leaves from young plants is consistent with their lower degree of structural development [42].

In the first experiment (Table 5), callogenesis began about one month after culture initiation, in both MS and WPM media, with slow biomass growth, particularly on WPM medium. Calli initially appeared red (especially on WPM) and later turned white to brown. During subsequent subcultures, calli progressively turned dark brown and showed reduced growth. This browning, likely due to oxidation [43], persisted even after separating newly formed calli from the explants. The browning of the material led to slower growth, with calli becoming shrivelled and hard. Tissue browning represented one of the main limitations during avocado callus establishment. This response is commonly reported in avocado in vitro culture and is mainly associated with the oxidation of phenolic compounds released after tissue wounding, which may generate brown products that become toxic to the explants and negatively affect regeneration and growth. Similar limitations have been described during avocado micropropagation, where browning caused by phenolic oxidation is considered one of the major factors reducing in vitro culture success. More generally, browning in plant tissue culture has been linked to phenolic accumulation and to the activity of enzymes involved in phenolic metabolism and oxidation, particularly polyphenol oxidase, peroxidase, and phenylalanine ammonia-lyase [44,45].

Consequently, to counteract this effect, a second trial was established (Table 5). In addition to MS and WPM, two alternative media (MSA and B5A) were introduced, in which NAA and K were present, alongside 2,4-D, to promote growth and delay tissue ageing [46]. The second experiment showed the same pattern of callogenesis, with a low response only on WPM, while the other media performed similarly (Table 5). Calli developed mainly as white, friable masses rather than red, although overall growth was slower than in the first experiment. As before, over time, the material tended to shrivel. Notably, the oxidised material on B5A medium, after 15 subcultures, proliferated into whitish-greyish-brownish biomasses, probably in response to stress conditions [43]; these calli regained vitality, appearing viable, hydrated, and actively growing.

The recovery observed in the second trial may reflect a gradual adaptation or selection of cell populations better able to tolerate in vitro stress conditions. In addition, the presence of NAA and K together with 2,4-D may have contributed to sustaining cell division and delaying tissue senescence, since auxin- and cytokinin-mediated regulation is central to callus induction and proliferation [47]. B5-based formulations may also be favourable for actively growing cell cultures, as this medium was originally developed for plant cell growth and contains a nitrogen/vitamin composition suitable for proliferating callus cultures (https://duchefa-biochemie.com/product/details/number/G0209?utm; accessed on 6 November 2026).

**Figure 1:**
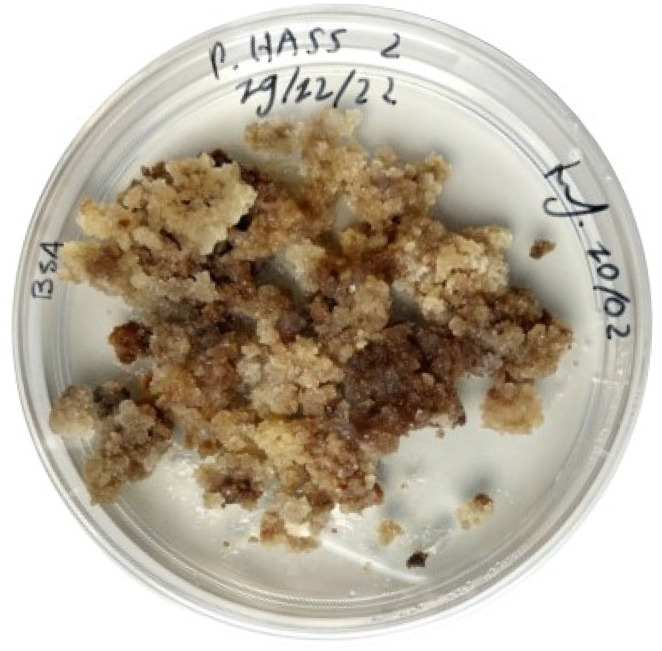
Calli in B5A medium.

Once the whitish-greyish-brownish proliferating biomass had replaced the oxidised material on B5A medium, these calli maintained a stable morphology and growth pattern throughout subsequent subcultures. Figure 1 shows calli after three years from the culture initiation.

### 3.2. Colourimetric Analyses

Phenolic compounds, particularly proanthocyanidins, are recognised as key contributors to the antioxidant properties of plant-derived materials, among which avocados, due to their strong radical scavenging capacity [12,46,48,49,50,51]. Given the relevance of these compound classes in modulating antioxidant activity, DPPH radical scavenging capacity was also evaluated alongside phenolic composition.

In this context, the total phenolic content (TPC), total proanthocyanidin content (TPcC), and DPPH radical scavenging activity were evaluated at three developmental stages (T14, T28, and T42) of the in vitro material. The experimental data are reported in Table 6. TPC showed moderate variation across sampling times, whereas TPcC exhibited a clear decreasing trend from T14 to T42 (162.78 to 113.74 µg/mL). A similar decrease was observed for DPPH radical scavenging activity; 6 μL of juice provides DPPH inhibition ranging from 52.91% to 43.63% (related respectively to juices harvested on the 14th and 42nd day of the growth cycle), giving the same inhibition as 2.29 and 1.91 μg of ascorbic acid, tested in the same conditions. These data may suggest a potential relationship between TPcC and antioxidant capacity.

**Figure 2:**
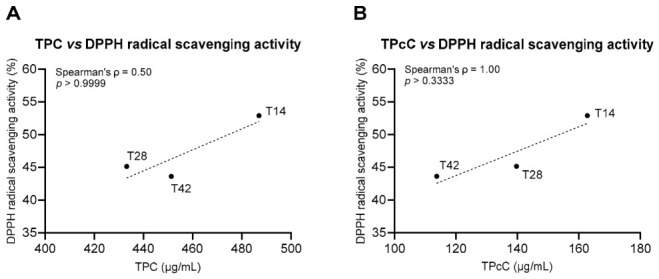
Scatter plots showing the relationship between phenolic compounds and DPPH radical scavenging activity. (**A**) Correlation between total phenolic content (TPC, μg/mL) and DPPH radical scavenging activity (%). (**B**) Correlation between total phenolic compound content (TpCC, μg/mL) and DPPH radical scavenging activity (%). Linear regression lines are shown for visual guidance. Data represent mean values (*n* = 2) obtained at three developmental stages (T14, T28, and T42). Spearman’s correlation coefficient (ρ) and *p*-values are reported in each panel.

Scatter plot analysis (Figure 2) confirmed these relationships. A moderate positive correlation was observed between TPC and DPPH radical scavenging activity (Spearman’s ρ = 0.50), whereas TPcC showed a strong positive correlation (ρ = 1.00), indicating a highly consistent monotonic relationship. Although these correlations were not statistically significant, the graphical representation provides clear visual evidence of a stronger association between TPcC and antioxidant activity compared to TPC.

Differences among sampling times were evaluated using the Kruskal–Wallis test (Table 7). No statistically significant differences were detected for any parameter. However, both TPC and TPcC showed a trend towards variation (*p* = 0.0667), whereas DPPH radical scavenging activity did not show statistically significant variation (*p* = 0.3333).

Although the number of replicates included in the final analysis was limited (*n* = 2), preliminary experiments consistently showed the same trends and confirmed the stability of the analysed parameters, supporting the reproducibility of the results. Furthermore, efforts were made to minimise sample and reagent consumption in line with sustainability considerations. Nevertheless, the reduced dataset limits the robustness of the statistical analyses, and therefore the results should be regarded as preliminary and exploratory. Within these limitations, the observed trend suggesting a closer association between TPcC and antioxidant activity than between TPC and antioxidant activity is plausible. Indeed, the possible role of TPcC in the antioxidant activity is consistent with the well-established role of condensed tannins as efficient radical scavengers. Their antioxidant properties are related to their chemical structure, including multiple hydroxyl groups and the ability to stabilise free radicals [46,48,52].

Recent studies on plant-derived extracts, including avocado matrices, have demonstrated that antioxidant activity is strongly influenced by the composition of phenolic subclasses rather than total phenolic content alone. For instance, avocado peel contains a complex mixture of polyphenols, including catechins and proanthocyanidins, which are closely associated with antioxidant capacity [12]. Similarly, extracts from avocado by-products have shown high antioxidant potential largely attributed to condensed tannins and flavan-3-ol derivatives [53,54].

Overall, these findings suggest that TPcC plays a role in determining DPPH radical scavenging activity during the in vitro growth cycle. Further studies, including a detailed phenolic profiling, would be required to confirm these observations since the antioxidant activity is often the result of the combined action of multiple classes of compounds, which may act additively or synergistically.

Following the correlation analysis between total phenolic content (TPC), total proanthocyanidin content (TPcC) and DPPH radical scavenging activity obtained in juices (expressed as µg/mL), the data were further normalised to fresh weight (FW) and dry weight (DW) of callus (Table 8). This conversion was performed to enable a more direct and reliable comparison with literature data, as most studies report phenolic contents in mg/g FW or DW. In this context, expressing results per unit of tissue (fresh and dry) allows decoupling the effect of extraction volume and water content, providing a more physiologically meaningful interpretation of metabolite accumulation.

When expressed on a fresh weight basis, TPC ranged from 0.32 to 0.36 mg/g FW, while TPcC ranged from 0.08 to 0.12 mg/g FW. These values are comparable to, or slightly higher than, those reported for avocado pulp, which typically contains 0.12–0.30 mg/g FW of phenolic compounds [12]. This finding indicates that avocado callus cultures are capable of accumulating appreciable amounts of phenolic metabolites despite their undifferentiated nature. On a dry weight basis (Table 8), TPC ranged from 6.48 to 7.98 mg/g DW, while TPcC ranged from 2.01 to 2.18 mg/g DW, values similar to and in some cases higher than those reported for avocado pulp too, and fall within the broader range described for avocado-derived biomasses. Within the avocado fruit, seeds and peel are generally recognised as the richest phenolic fractions due to their accumulation of flavonoids, proanthocyanidins, and other antioxidant metabolites, with peel often exhibiting the highest phenolic contents (>10 mg/g DW) [54,55,56,57,58]. However, comparisons with literature data should be indicative, as several studies report phenolic contents determined on optimised extracts obtained through targeted extraction procedures, including microwave-assisted extraction, which can substantially enhance the recovery of bioactive compounds and yield considerably higher values than those measured directly in plant tissues [59,60]. Consequently, these data are presented mainly to provide a comparative framework for the interpretation of the callus phytochemical profile, rather than as a strict comparison with the corresponding plant tissues.

The comparison between FW and DW highlights different aspects of phenolic metabolism. While TPC appears relatively stable on a fresh weight basis, it increases on a dry weight basis, suggesting a relative enrichment of phenolic compounds during development, likely due to water loss or changes in tissue composition. Conversely, TPcC shows a decreasing trend on a fresh weight basis, which parallels the decrease observed in DPPH radical scavenging activity. This supports the hypothesis that proanthocyanidins are key contributors to antioxidant capacity in this system.

These findings are consistent with previous studies demonstrating that antioxidant activity is more strongly associated with specific phenolic subclasses, such as condensed tannins, rather than total phenolic content alone [46,48,52].

### 3.3. LC–MS/MS Investigation

**Figure 3:**
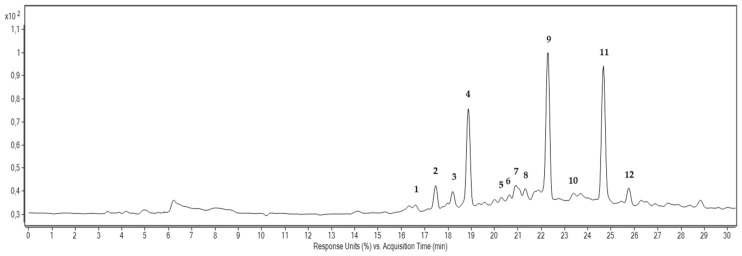
Juice chromatogram recorded at 325 nm showing the numbered peaks selected for preliminary LC-MS/MS characterisation. Tentative peak assignments are as follows: 1, galloyl derivative; 2, catechin-hydroxycinnamoyl derivative; 3, oxygenated flavanone/flavanonol-like compound; 4, 1-*O*-coumaroyl-2-*O*-rhamnosyl propanal; 5, catechin/epicatechin derivative; 6, apigenin-pentoside-like compound; 7, A-type procyanidin dimer; 8, apigenin-*O*-rhamnoside-like compound; 9, rhamnosyl dehydro-coumaroyl derivative; 10, glucosylated phenolic derivative; 11, nudiposide; 12, nd. nd = not determined.

The peaks detected in the chromatogram acquired at 325 nm were selected for the initial investigation because this wavelength provided one of the most informative chromatographic profiles among those monitored, revealing several almost well-resolved and relatively abundant UV-active metabolites. Consequently, the preliminary mass spectrometric characterisation was focused on these compounds in order to obtain a first overview of the principal secondary metabolite classes and biosynthetic pathways active in the analysed material juice. The chromatogram, acquired at 325 nm with the tentatively identified peaks numbered, is reported in Figure 3. Overall, the LC-MS/MS profile highlighted a predominance of phenolic metabolites, including galloyl derivatives, flavan-3-ols, proanthocyanidins, flavonoid glycosides and phenylpropanoid-like conjugates. This compositional pattern is consistent with previous reports on avocado non-edible tissues, particularly peel and seed, which are known to be significantly richer in phenolics than the pulp [51,57,58,61].

Particular attention was focused on compounds **4**, **9** and **11**, as these represented the most abundant UV-active metabolites detected in the chromatographic profile at 325 nm (Figure 3). Although the present study was not designed as a quantitative analysis, these compounds were initially prioritised to obtain a preliminary overview of the major secondary metabolic pathways active in *Persea americana* callus tissues.

Compound **4** (RT 18.85 min), detected as a formate adduct at *m*/*z* 427, generated major fragments at *m*/*z* 381 and 235. The UV spectrum, characterised by strong absorption in the low UV region together with a secondary band around 300 nm, was highly consistent with cinnamoyl-containing phenylpropanoids. Based on the molecular fragmentation reported by other authors, compound **4** was tentatively interpreted as a 1*-O*-coumaroyl-2-*O*-rhamnosyl propanal [62]. Compound **9** (RT 22.27 min) was among the most abundant UV-active metabolites detected in the chromatogram. The precursor ion at *m*/*z* 441 was interpreted as a formate adduct corresponding to a putative molecular ion at *m*/*z* 395, which fragmented into *m*/*z* 249, 161 and 143. The neutral loss of 146 Da suggested the occurrence of a deoxyhexose residue, likely rhamnose, whereas the fragments at *m*/*z* 161 and 143 were compatible with modified hydroxycinnamoyl-related moieties. The strong absorbance at 300 nm further supported the occurrence of a conjugated phenylpropanoid system. Although no exact spectral match was identified, the observed fragmentation behaviour and the UV spectrum overlapping with compound **4**, compound **9** was suggested to be a rhamnosyl dehydro-coumaroyl derivative. The occurrence of hydroxycinnamic acid derivatives and phenolic conjugates is already reported in avocado tissues [51]. Along with the previous compounds, compound **11** (RT 24.67 min) was observed as a formate adduct at *m*/*z* 597 with major fragments at *m*/*z* 551, 419, 233 and 153. Among the detected metabolites, compound **11** provided one of the strongest tentative annotations. Based on the parental ion (*m*/*z* 551), the observed fragmentation pattern, and the occurrence of structurally related lignans previously reported in plant materials [62], including the genus *Persea* [63], compound **11** was suggested to be nudiposide. In addition, nudisposide lignan has already been characterised in the polar fraction of avocado peel [64].

Besides the major UV-active metabolites, some additional compounds were tentatively associated with flavonoid and phenolic metabolism based on their *m*/*z* values, fragmentation patterns, and chromatographic elution order. A relatively polar metabolite (compound **1**) detected at RT 16.6 min (*m*/*z* 477 → 247 → 169 → 101) was tentatively assigned as a galloyl-related derivative due to the diagnostic fragment at *m*/*z* 169, commonly associated with gallic acid-derived structures [65]. Galloylated phenolics and related hydrolysable tannin metabolites have previously been reported in avocado peel and seed extracts, which are known to contain high levels of antioxidant phenolics [51]. At RT 17.4 min, compound **2**, detected as a formate adduct at *m*/*z* 481, yielding fragments at *m*/*z* 435, 289, and 163, was tentatively interpreted as a catechin-related hydroxycinnamoyl derivative. The simultaneous occurrence of the fragment ion at *m/z* 289, characteristic of flavan-3-ol substructures, together with the ion at *m*/*z* 163, compatible with hydroxycinnamoyl-related fragments, suggested a hybrid flavan-3-ol/phenylpropanoid structure. Similar catechin, epicatechin and procyanidin derivatives have previously been described in avocado seed and peel tissues [61]. Compound **3** (RT 18.19 min), detected as a chloride adduct at *m*/*z* 438 and generating fragments at *m*/*z* 403, 316 and 223, was tentatively associated with an oxygenated flavanone/flavanonol-like compound. Similar flavonoid glycosides and oxygenated flavonoids have been reported in avocado leaves and vegetative tissues [66]. Additional low-intensity flavan-3-ol-related metabolites were observed in the same chromatographic region, including compound **5** at *m*/*z* 431, fragmenting to *m/z* 289 and 224, tentatively interpreted as a catechin/epicatechin-related derivative. Likewise, a minor metabolite (**6**) detected as a formate adduct at *m*/*z* 447 and yielding fragments at *m/z* 401 and 269 was tentatively associated with an apigenin-pentoside [67]. At RT 20.9 min, compound **7** (*m*/*z* 575 → 417 → 289 → 221) was tentatively assigned as an A-type procyanidin dimer due to the formation of the characteristic flavan-3-ol fragment at *m*/*z* 289, in agreement with data reported for type-A proanthocyanidins [61,68]. The occurrence of procyanidins and catechin/epicatechin derivatives is consistent with previous phytochemical studies on avocado by-products, particularly seed and peel, where flavan-3-ols represent one of the dominant phenolic classes. In addition, one type of proanthocyanidin A dimer was already found in Colombian avocado seed and peel extract [61]. A further flavonoid-related metabolite (compound **8**) detected at RT 21.4 min, observed as a formate adduct at *m*/*z* 461, and fragmenting to *m*/*z* 415 and 269, was tentatively interpreted as an apigenin-*O*-rhamnoside-like compound. The fragment ion at *m*/*z* 269 was consistent with an apigenin-related aglycone linked to a rhamosyl moiety (146 *m*/*z*) [67]. At longer retention times, additional moderately hydrophobic phenolic derivatives were detected. Compound **10**, observed as a formate adduct at *m*/*z* 627 and yielding fragments at *m*/*z* 581, 419 and 153, was tentatively interpreted as a glucosylated phenolic derivative containing aromatic oxygenated substructures. Finally, compound **12** (RT 25.7 min), detected at *m*/*z* 613 and fragmenting to *m*/*z* 566, 399, 295 and 209, could not be confidently associated with any specific metabolite class. Nevertheless, its relatively late retention time and fragmentation behaviour suggested a moderately hydrophobic oxygenated phenolic derivative, possibly related to acylated flavonoid or phenylpropanoid metabolism.

Overall, the metabolite profile observed in this study resembled that previously reported for avocado peel, seed and vegetative tissues rather than the pulp, indicating that phenolics, including flavonoids, proanthocyanidins and hydroxycinnamoyl-derived conjugates, may represent major components of the investigated metabolite profile. The comparison with differentiated avocado tissues (pulp, peel, and seed) was intended to provide a compositional benchmark and facilitate the interpretation of the metabolomic data. Importantly, these tissues differ substantially from callus cultures in terms of cellular organisation, developmental status, and physiological function. Consequently, such comparisons should be regarded as indicative rather than demonstrative of biological equivalence, serving mainly to contextualise the metabolic features of the in vitro-derived biomass. Furthermore, the present work should be regarded as a preliminary mass spectrometric screening; the confidence associated with the proposed annotations varied among compounds, and annotations were based on fragmentation patterns, UV spectra, chromatographic behaviour and literature comparison. Consequently, all compound identifications remain tentative and further targeted MS/MS analyses will be necessary to achieve definitive structural characterisation.

### 3.4. Nutritional Analysis

To assess the potential of plant cell cultures as a source of agricultural products for human use, the nutritional profile of the Hass calli was analysed. The total content of moisture, ashes, carbohydrates, fibre, proteins and fats, expressed as a percentage of fresh weight (FW) and dry weight (DW), is shown in Table 9.

The compositional profile of Hass avocado callus differs markedly from that of the fruit and its derived tissues. In particular, lipid content in callus was significantly lower (0.9% FW; 15% DW-Table 6) than in fresh avocado pulp, which is well known to be rich in lipids (typically ≥15% FW) and characterised by a high proportion of nutritionally beneficial unsaturated fatty acids [3]. Lipid levels in callus were also lower than those reported for avocado seed and peel. Because avocado is widely recognised for its high lipid content and for the nutritional relevance of its fatty acid composition, the lipid fraction of the in vitro cultures was investigated in detail (Table 10).

Avocado is considered one of the richest lipid-containing fruits among horticultural crops, and its nutritional and functional value is mainly associated with the abundance of monounsaturated fatty acids, particularly oleic acid (C18:1 n-9), together with other bioactive lipid compounds [3]. Recent studies further confirmed that avocado lipids are characterised by a predominance of unsaturated fatty acids, especially oleic acid, followed by palmitic and linoleic acids, although their relative abundance may vary considerably according to cultivar, tissue type, environmental conditions and ripening stage [69,70]. The fatty acid analysis of the avocado in vitro cultures (Table 10) revealed a predominance of saturated fatty acids (56.62%), followed by monounsaturated fatty acids (35.32%) and polyunsaturated fatty acids (8.06%). This profile differs from that commonly reported for mature avocado mesocarp and avocado oil, where unsaturated fatty acids generally predominate. Comparative analyses of avocado cultivars demonstrated that oleic acid is normally the major fatty acid, frequently representing more than 50% of total lipids in several cultivars, whereas palmitic acid generally occurs at lower levels [69]. Moreover, recent molecular studies on avocado lipid metabolism showed that the balance between saturated and unsaturated fatty acids is highly cultivar-dependent [71]. In the present study, palmitic acid (C16:0) was the most abundant fatty acid, accounting for 30.81% of total fatty acids, while oleic acid (C18:1 n-9c) represented 29.82%. The relatively high palmitic acid content and the lower oleic acid proportion compared with conventional avocado tissues may reflect the physiological and metabolic characteristics of the cultured cells. In plant systems, palmitic acid is an important structural component of membrane lipids and plays a key role in cellular lipid metabolism [72]. The relatively high abundance observed in the present study may be associated with the actively proliferating and partially dedifferentiated nature of the in vitro cultures. Fatty acid composition in plant cells, grown in vivo and in vitro, is strongly influenced by cellular and environmental conditions, which can substantially alter the balance between saturated and unsaturated fatty acids through membrane lipid remodelling processes [73,74]. The elevated proportion of saturated fatty acids observed in the present cultures is therefore likely associated with the undifferentiated nature of the in vitro biomass rather than with the storage lipid composition typically found in mature avocado pulp. In addition, avocado fatty acid composition has been shown to be highly dynamic and responsive to physiological and environmental conditions, including maturation stage and tissue specialisation [69].

Among the polyunsaturated fatty acids, linoleic acid (C18:2 n-6c) was the predominant component, accounting for 6.49% of total fatty acids. This value is consistent with the lower ranges reported for avocado tissues and avocado oil in previous compositional studies [75]. α-Linolenic acid (C18:3 n-3) was detected only at low levels (0.60%), confirming that omega-3 fatty acids remain minor constituents of avocado lipid metabolism. Interestingly, minor saturated fatty acids such as lauric acid (5.07%), myristic acid (9.30%) and stearic acid (9.60%) contributed substantially to the overall saturated fraction, suggesting active membrane remodelling and culture-specific regulation of fatty acid biosynthesis.

Although quantitatively different from mature avocado mesocarp, the observed fatty acid composition confirms that the in vitro system preserves key aspects of avocado lipid metabolism. Importantly, these differences should not be interpreted as a limitation of the in vitro system, but rather as evidence of the adaptability and biotechnological potential of avocado cultured cells.

In contrast, carbohydrate content in callus (1.5% FW; 25% DW-Table 9) was comparable to the lower range reported for fresh avocado pulp (≈0.8–1.5% FW), while protein (12.8% DW) and fibre (35% DW) contents showed partial similarities with other avocado tissues. Notably, the protein content of callus appeared higher than that reported for avocado peel [76,77,78]. A defining feature of the callus was its very high moisture content (94% FW), exceeding typical values reported for avocado pulp (generally 60–80% FW depending on cultivar and ripening stage), which is consistent with the highly hydrated and undifferentiated nature of in vitro plant cell cultures [79,80]. Consequently, the caloric value of fresh callus was low (21 kcal/100 g FW-Table 9), whereas on a dry-weight basis (350 kcal/100 g DW-Table 9), it reflects the concentration of macronutrients. Overall, unlike the lipid-rich storage profile of avocado pulp, the callus resembles a metabolically active plant biomass, characterised by high water, fibre, and protein content, reduced lipid accumulation, and a relative enrichment in structural carbohydrates and mineral components. Given that the high moisture content of the callus biomass may limit the practical nutritional contribution of proteins and fibres under direct consumption scenarios, these components may become nutritionally more relevant in dried, concentrated, or processed biomass products. In light of the well-documented nutritional and functional properties of avocado tissues and by-products, the comparison with avocado pulp, peel, and seed, undertaken to contextualise the nutritional profile of the in vitro-derived biomass within the compositional diversity of the species, provides a useful framework for evaluating the potential relevance of callus biomass.

The results obtained in the present study provide a preliminary indication that avocado cell cultures may represent a promising source of bioactive compounds and nutrients, as already reported in the few available studies in this research area. However, these findings should be considered exploratory, and substantial further research is required before any practical food application can be envisaged. In particular, comprehensive investigations on biomass composition, bioavailability, absorption, distribution, metabolism and excretion (ADME), toxicological safety, and long-term effects are needed. Moreover, compliance with the regulatory framework governing novel foods in the European Union will be essential. Future studies should also address sensory properties, technological performance in food matrices, and consumer acceptance. Therefore, although the present results support the potential of plant cell cultures as a sustainable platform for the production of value-added ingredients, additional multidisciplinary research is necessary to validate their suitability for food applications and support their eventual market introduction [23,24,25,81].

## 4. Conclusions

Avocado callus cultures showed a phenolic-rich profile, as indicated by both colourimetric assays and LC–MS screening, confirming the presence of diverse classes of secondary metabolites, including flavonoids, proanthocyanidins and phenylpropanoid derivatives. The overall composition differed markedly from fruit pulp, reflecting a metabolically active tissue with high moisture content and reduced lipid accumulation. Despite the preliminary nature of the analysis, the results consistently highlight the biochemical potential of this in vitro biomass. In addition, colourimetric tests supported the abundance of antioxidant-related compounds, reinforcing the relevance of the detected phenolic fraction. The successful induction and initial characterisation of avocado callus cultures represents a first step toward the development of avocado cell-based biotechnological platforms. Future studies should focus on establishing cell suspension cultures and, if possible, their scale-up in bioreactor systems to evaluate biomass production and metabolite yields under controlled conditions. Beyond contributing to biodiversity preservation, such platforms could offer enhanced process control, reproducibility, and independence from seasonal and environmental fluctuations. These characteristics are among the key advantages inherent to plant cell culture systems and underpin their potential as future alternative production platforms. Although substantial research is still required before industrial implementation can be considered, the present study lays the foundation for the future exploitation of avocado in vitro-derived biomass as a potential source of high-value compounds for food and nutraceutical applications.

## Figures and Tables

**Table 1:** Summary of preliminary experiments’ sterilisation procedure.

	Sterilisation (min)	
Material	EtOH (80%)	NaClO (15%)
peel	2	10
endosperm	2	6
leaf	2	10

**Table 2:** Summary of media tested in the preliminary experiments.

Medium	Basal Medium	2,4-D mg/L	NAA mg/L	K mg/L
B5-NAK	B5 [31]		1	1
WPM-NAK	WPM [32]		1	1
B5-DK	B5	1		1
WPM-DK	WPM	1		1
LTV	LTV [33]	1		
B5	B5	1		
WPM	WPM	1		
MS	MS [34]	1		

**Table 3:** MSA and B5A media’s plant growth regulator balances.

Medium	Basal Medium	2,4-D mg/L	NAA mg/L	K mg/L
**MSA**	MS	1.3	0.25	0.25
**B5A**	B5	1.3	0.25	0.25

**Table 4:** Callogenetic response of leaf explants in the media tested.

Medium	Callogenetic Response *
B5-NAK	−
WPM-NAK	−
B5-DK	+/−
WPM-DK	+/−
LTV	+
B5	+
WPM	++
MS	++

* Callogenetic response: ++ high; + medium-high; +/− low; − very low.

**Table 5:** Summary of the two trials.

	Harvesting Period	Contamination (%)	Callogenesis (%)			
			MS	WPM	MSA	B5A
I	June 2022	3	59	57	-	-
II	December 2022	5	44	17	48	49

**Table 6:** Experimental data of the total phenolic content (TPC), total proanthocyanidin content (TPcC), expressed as µg/mL, and DPPH radical scavenging activity (DPPH %), expressed as percentage. Data reported as mean ± SD, *n* = 2.

Time Point	TPC (µg/mL)	TPcC (µg/mL)	DPPH (%)
T14	486.97 ± 8.05	162.78 ± 9.52	52.91 ± 1.15
T28	433.17 ± 5.06	139.73 ± 5.86	45.15 ± 0.10
T42	451.33 ± 1.38	113.74 ± 8.78	43.63 ± 1.84

**Table 7:** Kruskal–Wallis test results for total phenolic content (TPC), total proanthocyanidin content (TPcC), and DPPH radical (DPPH) scavenging activity measured across the three developmental stages (T14, T28, and T42). Reported values correspond to the *p*-values obtained for each parameter. Data were derived from two replicates (*n* = 2) per developmental stage (T14, T28, and T42).

Parameter	*p*-Value
TPC	0.0667
TPcC	0.0667
DPPH	0.3333

**Table 8:** TPC (total phenolic content) and TPcC (total proanthocyanidin content) expressed as mg/g of fresh weight (FW) and dry weight (DW).

Time	TPC (mg/g FW)	TPC (mg/g DW)	TPcC (mg/g FW)	TPcC (mg/g DW)
T14	0.36	6.48	0.12	2.17
T28	0.32	6.75	0.10	2.18
T42	0.32	7.98	0.08	2.01

**Table 9:** Moisture (only for fresh weight), ashes, carbohydrates, fibre, proteins, fats and kcal expressed as percentage of fresh weight (% FW) and dry weight (% DW).

	% FW	% DW
moisture	94.0	-
ashes	0.7	11.8
carbohydrates	1.5	25.0
fibre	2.1	35.0
proteins	0.7	12.8
fats	0.9	15.0
kcal	21	350

**Table 10:** Fatty acid composition (%) of avocado fresh calli, including the relative abundance of saturated, monounsaturated, and polyunsaturated fatty acids, together with the overall distribution of lipid classes and omega-3/omega-6 fatty acid ratios.

	(%)
**Saturated Fatty Acids**	
C 4:0 Butyric	<0.01
C 6:0 Caproic	<0.01
C 8:0 Caprylic	<0.01
C 10:0 Capric	<0.01
C 11:0 Undecanoic	<0.01
C 12:0 Lauric	5.07
C 13:0 Tridecanoic	0.10
C 14:0 Myristic	9.30
C 15:0 Pentadecanoic	0.95
C 16:0 Palmitic	30.81
C 17:0 Heptadecanoic	0.57
C 18:0 Stearic	9.60
C 20:0 Arachidic	0.22
C 21:0 Heneicosanoic	<0.01
C 22:0 Behenic	<0.01
C 23:0 Tricosanoic	<0.01
C 24:0 Lignoceric	<0.01
**Monounsaturated Fatty Acids**	
C 14:1 Myristoleic	0.82
C 15:1 Pentadecenoic	<0.01
C 16:1 Palmitoleic	1.93
C 17:1 Heptadecenoic	0.19
C 18:1 n-7 Oleic	0.95
C 18:1 n-9c Oleic	29.82
C 18:1 n9-t11 Oleic	1.44
C 20:1 Eicosenoic	0.17
C 22:1 n-9 Erucic	<0.01
C 24:1 Nervonic	<0.01
**Polyunsaturated Fatty Acids**	
C 18:2 n-6c Linoleic	6.49
C 18:2 t (isomers) Linoleic	0.29
Conjugated Linoleic Acid (CLA total)	0.29
C 18:3 n-3 Linolenic	0.60
C 18:3 n-6 Linolenic	0.02
C 18:3 t (isomers) Linolenic	0.07
C 18:4 n-3 Octadecatetraenoic	<0.01
C 20:2 n-6 Eicosadienoic	0.06
C 20:3 n-3 Eicosatrienoic	<0.01
C 20:3 n-6 Eicosatrienoic	0.07
C 20:4 n-3 Arachidonic	0.02
C 20:4 n-6 Arachidonic	0.12
C 20:5 n-3 Eicosapentaenoic (EPA)	0.03
C 22:2 n-6 Docosadienoic	<0.01
C 22:5 n-3 Docosapentaenoic (DPA)	<0.01
C 22:6 n-3 Docosahexaenoic (DHA)	<0.01
**Summary**	
Total Saturated Fatty Acids	56.62
Total Monounsaturated Fatty Acids	35.32
Total Polyunsaturated Fatty Acids	8.06
Trans Fatty Acids	1.80
Omega-3 Fatty Acids (Total)	0.65
Omega-6 Fatty Acids (Total)	7.05
Omega-3/Omega-6 Ratio	0.09

## Data Availability

The original contributions presented in this study are included in the article. Further inquiries can be directed to the corresponding author.
